# Effectiveness and safety of warm needle acupuncture on insomnia: Protocol for a systematic review and meta-analysis

**DOI:** 10.1097/MD.0000000000013598

**Published:** 2018-12-21

**Authors:** Baishu Chen, GangYu Zhang, Cuiling Liu, QianYing Chen, MingJia Zhang, JianHao Li, Peng Zhou, Wei Fu, Meiling Zhu

**Affiliations:** a Baoan Hospital of Traditional Chinese Medicine in Shenzhen, Shenzhen; b Guangzhou University of Chinese Medicine, Guangzhou, China.

**Keywords:** insomnia, protocol, systematic review, warm needle acupuncture

## Abstract

**Background:**

Warm needle acupuncture (WNA) combines acupuncture and moxibustion, which is an integral part of the acupuncture therapy. Insomnia is a common sleep disorder, which affects sub-healthy people and patients with chronic disease. The clinical practice indicates that WNA has a therapeutic effect on insomnia. Here we will provide a protocol to explore the effectiveness and safety of WNA for insomnia.

**Methods:**

We will search the randomized controlled trails (RCT) literatures of WNA for insomnia in 9 electronic databases, including 5 English databases [PubMed, Web of Science, EMBASE, the Cochrane Central Register of Controlled Trials (Cochrane Library), and WHO International Clinical Trials Registry Platform (TCTRP)] and 4 Chinese databases [Chinese National Knowledge Infrastructure (CNKI), Chinese VIP Information, Wanfang Database, and Chinese Biomedical Literature Database (CBM)]. Sleep quality value of the patient will be considered as the primary outcome and the secondary outcome will include biochemical, indicators total scores on the insomnia severity index, quality of life, adverse events caused by WNA, and changes of symptom in Traditional Chinese Medicine. The selection of the studies will be performed by EndnoteX7 software. All analyses will be conducted by using RevMan software V5.3.

**Result:**

This study will provide a rational synthesis of current evidences for warm needle acupuncture on insomnia.

**Conclusion:**

The conclusion of this study will provide evidence to judge the effectiveness and safety of WNA on insomnia.

**Registration:**

PROS-PERO CRD42018112645

## 1 Introduction

Insomnia is a common disease characterized by difficulty falling and/or staying asleep, and accompanied by irritability or fatigue during wakefulness.^[1]^ It is widely reported that insomnia is one of the most extensive mental disorders which the incidence rate is estimated to be about 10%.^[2]^ Insomnia can have serious influences on patients’ health and quality of life.^[3]^ Moreover, insomnia is a risk factor of psychiatric disorder,^[4]^other medical disorders,^[5]^ and increases costs of health care.^[6]^ The etiology and pathophysiology of insomnia involve a wide range of genetic, environmental, behavioral, and physiological factors that ultimately lead to over-excitement without falling asleep.^[6]^ Effective treatment for insomnia includes non medicine methods, medicines, or both.^[3,7]^ There are many non medicinal methods such as acupuncture and other therapies. One of the most common treatments is cognitive behavioral therapy for insomnia (CBT-I). The CBT-I is an effective way and harm related to CBT-I is rare.^[8]^ Unfortunately, many people with insomnia cannot benefit from this treatment because of problems with accessibility and cost effectiveness.^[7,9]^ In clinical practice, many sedatives and hypnotic can be used to treat insomnia.^[10,11]^ However, the use of these drugs in insomnia patients is challenging due to the side effects like tolerance and dependence. They are under a high risk of feeling drowsy, lightheaded, and dizzy.^[10]^ Furthermore, these medicines have the potential for fractures from falls, dementia symptoms, depression that gets worse over time, and impaired driving.^[10]^ Thus, these medicines should only be given for a short time, usually 4 to 5 weeks.^[12]^ In general neither of the methods is perfect and both have their own defects. Therefore, numerous research studies have been carried out on this topic to reduce the symptoms of insomnia in patients with efficiency and safety.

Warm needle acupuncture (WNA) is a combinatorial therapy of moxibustion and acupuncture, which is one of the most popular traditional Chinese medicine (TCM) techniques used routinely in Asian countries. Effects of Warm acupuncture consist of acupuncture effect, warming effect, and acupoint-specific stimulation.^[13]^ Through a complex mechanism of multi-system, multi-channels such as blood circulation, nervous system, and immune function, it can achieve the goal of regulating the body and treating diseases.^[14]^ The WNA has the functions of enhancing immunity, regulating blood circulation, regulating voxels, and preventing diseases.^[13]^ Some clinical trials have found that WNA has a significant impact on insomnia. In addition, it has the advantages of safe, reliable, and is easy to use without toxic and side effects.^[15]^ However, relationships between WNA and insomnia have not been revealed clearly. Thus the purpose of this review is to summarize clinical researches on WNA for insomnia and findings of this review will be reliable within evidence of clinical studies. This review only focuses on the effects of warm acupuncture on insomnia rather than other effective treatments.

## 2 Methods

### 2.1 Study registration

The protocol has been registered on the International Prospective Register of Systematic Reviews (PROSPERO) (registration number, CRD42018112645) basing on the Preferred Reporting Items for Systematic Reviews and Meta-Analyses Protocols (PRISMA-P) statement guidelines^[16]^ on 31^th^ Oct 2018.

### 2.2 Inclusion criteria for study selection

#### 2.2.1 Types of studies

All available randomized controlled trials (RCTs) on WNA for insomnia will be included. Others such as retrospective study, case report, review, and studies which uses inappropriate random sequence generation methods will be excluded. Language will be restricted to Chinese and English.

#### 2.2.2 Types of participants

We will include studies on patients that have been diagnosed as insomnia by clinicians based on Diagnostic and Statistical Manual of Mental Disorders, 4th edition (DSM-IV),^[17]^ International Statistical Classification of Diseases and Health Related Problems, 10th revision (ICD-10),^[18]^ and International Classification of Sleep Disorders (ICSD).^[19]^ There will be no restriction on age, gender, ethnicity, and profession.

#### 2.2.3 Types of interventions

The purpose of the study is on clinical trials of WNA for insomnia. Studies applied WNA in the experimental group will be included. The WNA combined with other therapies will be excluded if the efficacy of WNA cannot be clarified in the combined therapy. The therapeutic intervention of controlled group can be conventional acupuncture, electro-acupuncture, auriculo-acupuncture or pharmcological therapy (such as benzodiazepines).

#### 2.2.4 Types of outcome measures

##### 2.2.4.1 Primary outcome

The primary outcome is the sleep quality values according to the Pittsburgh Sleep Quality Index (PSQI) which is comprised of 19 self-rated items and 5 other-rated items.^[20]^

##### 2.2.4.2 Secondary outcomes

- Insomnia severity according to the Insomnia Severity Index (ISI).^[21]^
- Quality of life measured by validated assessment tools such as the 36-Items Short Form Health Survey (SF-36).^[22]^
- Syndrome according to standards for assessing TCM.
- Adverse events caused by WNA, such as dizziness, nausea, vomiting, weariness, etc.

### 2.3 Search methods for study identification

#### 2.3.1 Electronic searches

To identify relevant randomized controlled trials, 5 English databases [PubMed, Web of Science, EMBASE, the Cochrane Central Register of Controlled Trials (Cochrane Library), and WHO International Clinical Trials Registry Platform (TCTRP)] and 4 Chinese databases [Chinese National Knowledge Infrastructure (CNKI), Chinese VIP Information, Wanfang Database, and Chinese Biomedical Literature Database (CBM)] will be searched comprehensively from their inception to October 2018 by 2 independent researchers (and GY). The search items will be used as follows: insomnia, WNA, and RCTs. The search terms with the equivalent English meaning will also be used in Chinese databases. The detailed search strategies in PubMed are provided in Table 1 and will be used similarly in other databases.

**Table 1:**
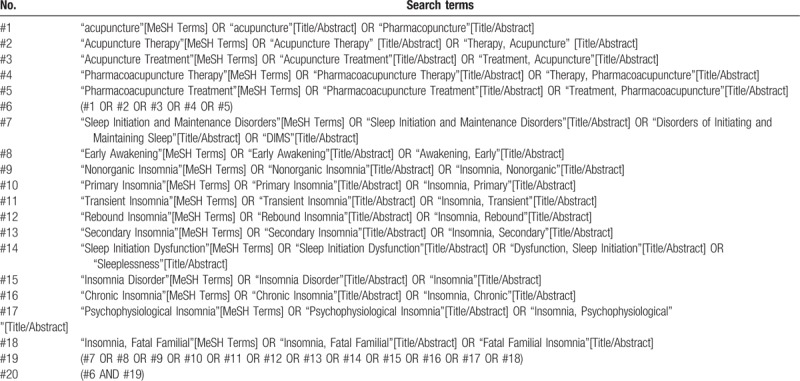
Search strategies for the PubMed.

#### 2.3.2 Searching other resources

Relevant systematic review or meta-analysis of RCTs will be electronically searched. Moreover, we will filter relevant medical journals and magazines to identify literature which is not included in the electronic databases.

### 2.4 Data collection and analysis

#### 2.4.1 Selection of studies

**Figure 1:**
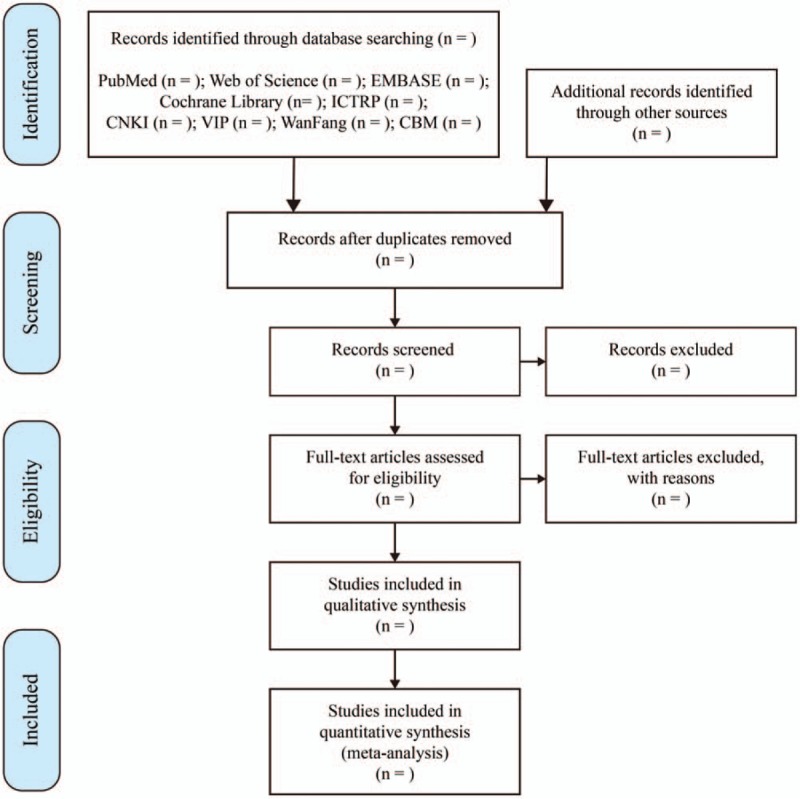
Flow chart of the study selection procedure.

Two researchers will import the relevant studies obtained from the databases mentioned above into EndnoteX7, a literature management software. After removing duplicates, 2 researchers will independently evaluate the titles and abstracts of the searched studies and exclude the significantly unqualified literature. Later, the full text of the remaining studies will be read carefully and selected according to the inclusive criteria. Any different opinions generated between the 2 reviewers will be resolved through discussion. When consultation fails to reach an agreement, the third reviewer will step in and provide arbitration. The study selection procedure is shown in a flow chart according to PRISMA guidelines^[23]^ (Fig. 1).

#### 2.4.2 Data extraction and management

After reading the full texts of each included articles, 2 independent researchers will extract the data via a standardized data collection form. The general information such as 1st author, country, year of publication, design of study, basic condition of the patient, sample size and number of dropouts, duration of follow-up, details of intervention, outcome measures, and adverse events associated with WNA will be extracted and recorded. If the data are ambiguous or insufficient, we will contact the authors to request detailed information via e-mail or telephone. Any divergence on data extraction will be discussed and judged by the 2 reviewers. The 3rd reviewer will check the final results of the data extraction and provide arbitration for further disagreements.

#### 2.4.3 Assessment of risk of bias in included studies

To evaluate the risk of bias of all included studies, 2 independent review authors will use the Cochrane Collaboration's risk of bias tool^[24]^ to assess the following domains: random sequence generation, allocation concealment, blinding to participants, personnel and outcome, incomplete outcome data, selective reporting, and other biases. Any discrepancies in the assessment of risk of bias will be resolved by discussion and an arbiter will be consulted if it is necessary. Ultimately, the quality of the studies will be divided into 3 levels: “low risk of bias”, “high risk of bias”, and “unclear risk of bias”.

#### 2.4.4 Measures of treatment effect

For continuous variables, mean difference (MD) will be used to evaluate the extracted data. For dichotomous variables, rate ratio (RR) will be applied to analyze. The confidence intervals (CI) for both continuous and dichotomous variables will be set to 95%.

#### 2.4.5 Dealing with missing data

For insufficient or missing trial data, we will try to contact the corresponding author by telephone or e-mail. If the missing data cannot be supplied or failure to contact the author, we will perform a limited analysis based on the available data and discuss the potential impact of the missing data.

#### 2.4.6 Assessment of heterogeneity

We will evaluate the heterogeneity with the use of *I*^*2*^ values in accordance with the Cochrane Handbook (0–40%, might not be important, 30–60%, may represent moderate heterogeneity, 50–90%, may represent substantial heterogeneity, and 75–100% may represent considerable heterogeneity). If the heterogeneity among trials is significant (***I***^***2***^≥50%), the random effects model will be selected and further subgroup analysis will be performed to investigate the possible causes of heterogeneity. Conversely, if an ***I***^***2***^ values less than 50%, we will choose the fixed effect model.

#### 2.4.7 Assessment of reporting bias

The presence of reporting bias will be evaluated by funnel plots when studies included in the review are sufficient (more than 10 trials). It will be considered that the reporting bias is existing and the reliability is low if the points on both sides of the funnel plot are dispersed and asymmetrical. Conversely, if the points on either side of the funnel plot are symmetrically distributed in substantial, the reporting bias will be considered as non-existent and the result is reliable.

#### 2.4.8 Data synthesis

All analyses will be conducted by using RevMan software (V5.3, The Nordic Cochrane Centre, The Cochrane Collaboration, Copenhagen, Denmark). We will select a random effects model or fixed effects model to merge the primary and secondary outcome indicators in accordance with the results of heterogeneity test. The fixed effects model will be applied for data synthesis of low heterogeneity (***I***^***2***^ < 50%) while the random effects model will be conducted if the heterogeneity is significant (***I***^***2***^≥50%). It is considered that differences are statistically significant if the results of Z test show that *P* value is less than 0.05, and the 95% CI does not contain 0 (for continuous variables) or the 95% CI does not contain 1 (for dichotomous variables).

#### 2.4.9 Subgroup analysis

If heterogeneity is evaluated as significant (*I*^*2*^≥50%) and the trials included are adequate, we will perform a subgroup analysis to explore the potential source of the heterogeneity according to the difference in participant characteristics, interventions, controls, and outcome measures.

#### 2.4.10 Sensitivity analysis

According to sample size, methodological quality, and the effect of missing data, sensitivity analysis will be carried out to identify the quality and robustness of the meta-analysis result when the outcome analyses involve a large degree of heterogeneity.

#### 2.4.11 Grading the quality of evidence

We will evaluate the quality of evidence and rate it into four levels: high, moderate, low, or very low in accordance with the Recommendations Assessment, Development and Evaluation (GRADE) guidelines.^[25]^

#### 2.4.12 Ethics and dissemination

Ethical approval will not be necessary because the data included in our study are derived from published literature and are not linked to individual patient data. The systematic review providing implication of the effectiveness and safety of WNA for insomnia will be published in a peer-reviewed journal or conference presentations.

## 3 Discussion

Insomnia is the most prevalent sleep disorder and an independent risk factor for several diseases, such as diabetes mellitus, cardiovascular disease, affective disorder, etc.^[26]^ The pharmacotherapies for insomnia can cause adverse effects, while placebo effects of the pharmacological therapy should not be neglected and the ideal medication is undiscovered at present.^[27]^ Therefore, it is necessary to search for alternative therapy with higher effective rate and less side effects.

Acupuncture is an extensively accepted alternative therapy around the world. As an indispensable part of acupuncture, WNA is widely applied in clinics. It is reported that WNA is superior to regular acupuncture on relieving pain, improving the blood circulation, stimulating metabolism of local tissue, etc.^[28,29]^ However, the evidence of efficacy and safety of WNA is insufficient and the underlying mechanisms remain largely unknown. Therefore, it is imperative to perform a systematic review and meta-analysis of available literature to evaluate the clinical efficacy and safety of WNA on insomnia objectively.

To the best of our knowledge, it will be the 1st systematic review and meta-analysis on WNA in the treatment of insomnia. Firstly, the results of this review will provide objective statistics for further researches on WNA. Secondly, the results will offer reliable references for clinicians and patients in the treatment of insomnia with WNA. Thirdly, the results may introduce an alternative therapy of insomnia to policy makers to decrease the burden of public health.

## Author contributions

Meiling Zhu conceived the study idea. Baishu Chen were responsible for the design of this systematic review. GangYu Zhang, QianYing Chen, and Peng Zhou contributed to the data analysis plan. Cuiling Liu and MingJia Zhang drafted the manuscript and JianHao Li, Wei Fu edited. All authors provided feedback and approved the final manuscript.

**Conceptualization:** Baishu Chen, Meiling Zhu.

**Methodology:** Cuiling Liu, QianYing Chen.

**Resources:** MingJia Zhang.

**Software:** JianHao Li.

**Writing – original draft:** GangYu Zhang.

**Writing – review & editing:** Peng Zhou, Wei Fu.
